# Improving the Storability of Cod Fish-Burgers According to the Zero-Waste Approach

**DOI:** 10.3390/foods10091972

**Published:** 2021-08-24

**Authors:** Flavia Dilucia, Valentina Lacivita, Matteo Alessandro Del Nobile, Amalia Conte

**Affiliations:** Department of Agricultural Sciences, Food and Environment, University of Foggia, Via Napoli, 25, 71121 Foggia, Italy; flavia.dilucia@unifg.it (F.D.); valentina.lacivita@unifg.it (V.L.); amalia.conte@unifg.it (A.C.)

**Keywords:** antimicrobial activity, fish storability, prickly pear cactus, by-products, sustainable approach, zero-waste

## Abstract

This research explored the potential of the zero-waste concept in relation to the storability of fresh food products. In particular, the prickly pear (*Opuntia ficus-indica*) peel (usually perceived as a by-product) and the pulp were dehydrated, reduced in powder, and used as food additives to slow down the growth of the main spoilage microorganisms of fresh cod fish burgers. The proportion between peel and pulp powder was such as to respect the zero-waste concept. The antibacterial activity of the peel and pulp in proper proportion was first assessed by means of an in vitro test against target microorganisms. Then, the active powder was added at three concentrations (i.e., 2.5 g, 7.5 g, and 12.5 g) to cod fish burgers to assess its effectiveness in slowing down the microbial and sensory quality decay of burgers stored at 4 °C. The results from the in vitro test showed that both the peel and pulp were effective in delaying microbial growth. The subsequent storability test substantially confirmed the in vitro test results. In fact, a significant reduction in growth rate of the main fish spoilage microorganisms (i.e., *Pseudomonas* spp., psychrotrophic bacteria, and psychrotolerant and heat-labile aerobic bacteria) was observed during 16 days of refrigerated storage. As expected, the antimicrobial effectiveness of powder increased as its concentration increased. Surprisingly, its addition did not affect the sensory quality of fish. Moreover, it was proven that this active powder can improve the fish sensory quality during the storage period.

## 1. Introduction

Over the past few years, the amount of food waste produced and lost through the supply chain has become a severe problem for the world, causing nutrient loss, climate changes due to the production of greenhouse gas, and losses of resources like water and cultivated land [1].

One of the approaches suggested to reduce this global concern is the concept of zero-waste, a philosophy that prompts to find a way for all products to be recycled, so that no kind of waste will be sent to landfills or incinerators [2]. Large quantities of waste are generated during processing of fruit and vegetables. The by-products (seeds, peels, and pomace) represent about 25–30% of them [3]. These are discarded in landfill or incinerated, creating serious environmental complications and economic expenses, used for animal feeding or for the production of biogas or bio-fertilizers, but they possess many bioactive compounds, like phenolic acids, flavonoids, vitamins, as well as antioxidant and antimicrobial activity. As a consequence, these by-products can be applied to food fortification as a source of valuable bio-components, as well as for food packaging to enhance film performance [4,5].

Prickly pear cactus, *Opuntia ficus-indica* (L.) *Miller*, a Cactateae, is a tropical or subtropical plant, mostly deriving from Mexico, but also found throughout the American continent, over the Mediterranean basin and in southern Spain [6]. The world production of prickly pear is about 1 million tons per year [7]. Based on the cultivar and ripening stage, prickly pear consists of peel (accounts for 33 to 55%), pulp (accounts for 45 to 67%) and seeds (accounts for 2 to 10%) [7,8]. It is widely used as fresh fruit or for manufacturing fruit juice [9] and alcoholic beverages [10]. The peel, that represents the major waste in prickly pear, is considered an agricultural by-product, even though it is a source of dietary fibers, proteins and antioxidant compounds [7]. Additionally, both the fruit and the peel are rich in polyphenolic compounds that show biological and antimicrobial activity against different microorganisms [9,11,12,13]. A few applications of prickly pear by-products to foods are available. In particular, Palmeri et al. [12] used whole prickly pear extract to improve the shelf life of sliced beef. Chougui et al. [14] studied the application of the hydro-ethanolic extract of prickly pear peel to preserve margarine. In addition, some authors have used prickly pear peel powder as a functional ingredient to formulate bread and biscuits [7,15]. To the best of our knowledge, no zero-waste production has been proposed with prickly pears. However, giving the potential properties of this fruit, it can be supposed that the efforts in food science to recycle prickly pear by-products could gain industrial relevance.

Seafood products are dynamic and prone to innovation, with fish being highly appreciated but very often considered a time-consuming food to prepare. Therefore, fish-based food, as fish burgers, represent a valid solution to accomplish consumer preference with products that have high nutritional value and are also very convenient, being ready-to-cook [16,17]. Generally, raw materials, processing technologies, storage conditions, enzymes of fish, and microflora are mainly responsible for fresh fish unacceptability [18]. Even though various preservation strategies are proposed in the literature to preserve seafood products [19,20,21], the most diffused approach for fresh fish burgers is based on the adoption of natural compounds, properly encapsulated or combined with modified atmosphere conditions [16,17,22,23,24] or enclosed in edible films [25].

Therefore, in the perspective of a more sustainable food production, the current study, for the first time explored the possibility to adopt all parts of prickly pear fruit for preserving fish burger quality during storage. Specifically, the pulp and peels of prickly pears were dehydrated, ground and included in the fish formulation according to the zero-waste approach, i.e., without producing any waste. The study demonstrated that the newly developed fish burgers, enriched with bioactive compounds from prickly pears, not only exerted good antimicrobial activity against the traditional fish spoilage, but they were also more appreciated than the control sample in terms of sensory quality, thus demonstrating the feasibility of the sustainable approach.

## 2. Materials and Methods

### 2.1. Prickly Pears

Red prickly pear fruits (*Opuntia ficus-indica*, (L.) *Miller*, cultivar *Sanguigna*) were kindly provided by a local dealer (Manfredonia, Puglia, Italy), in mid-September 2020. At the laboratory, the prickly pears were washed several times with water to remove any kind of residue and dipped into chlorinated water (20 mL·L^−1^) for 1 min. Then, the fruits were rinsed with water and air dried. After that, the peel was manually separated from the pulp using a knife and cut into strips, while the pulp, along with the seeds, was cut into small pieces. The peel and pulp were dried at 37 °C for a week using a vacuum dehydrator (Melchioni-Babele, Milan, Italy), and then milled in a lab-grinder to obtain a fine powder (500 micrometers). The two powders (pulp moisture 11.24 ± 0.76% and peel moisture 19.04 ± 0.20%), were stored separately in polyethylene bags at −20 °C until their use. In particular, 19520.1 g of fresh prickly pears was processed; they were composed by 8429.29 g of peel and 11090.81 g of pulp. They were dehydrated and 3049.89 g of dry prickly pear powder was obtained, composed by 1739.72 g pulp, and 1310.17 g of peel. To use all parts of prickly pears, according to the zero-waste approach, for 1 g of prickly pear powder, 0.57 g of pulp and 0.43 g of peel were used.

### 2.2. Fish Burger Formulation

Frozen cod (*Gadus morhua*) fillets were purchased from a local market (Ipercoop, Foggia, Italy) and were minced with a knife in small pieces. The formulation of the control burger and of the three active samples enriched with the prickly pear powder (named CNT, ACT-2.5, ACT-7.5 and ACT-12.5), is described in Table 1. The fish burgers were obtained by first mixing the flours (i.e., potato starch and potato flakes), salt, and the prickly pear powder, then adding the extra-virgin olive oil, and finally adding the minced cod fillets. Thereafter, the prepared mixture was shaped by means of a metal shaper to obtain fish burgers of 1.5 cm thick, and 5 cm in diameter. Three independent replicates were prepared for each formulation. All investigated burgers were packed into polyethylene bags, sealed and stored at 4 °C.

### 2.3. Prickly Pear Powder Antibacterial Activity: In Vitro Test

The antimicrobial activity of prickly pear peel and pulp powders was evaluated by an in vitro test. To this aim, two strains of *Pseudomonas* spp. (*P. fluorescens* and *P. putida*) were used as target microorganisms, stored at −20 °C as stock cultures. The exponentially growing cultures were obtained in Plate Count Broth (PCB, tryptone 5 g/L, glucose 1 g/L and yeast extract 2.5 g/L, Oxoid) at 25 °C for 24 h. After that, a cocktail of the two strains was diluted with 0.9% NaCl to obtain approximately 10^3^ CFU/mL. The PCB inoculated with the microbial cocktail was placed in several tubes. Every tube also contained 2.5% and 5% of prickly pear peel powder, 2.5%, and 5% of prickly pear pulp powder for the active samples, and no powder was added for the control one. The pulp and the peel allowed to settle in each tube. All tubes were incubated at 25 °C for 72 h. Microbiological analyses were performed after 0, 4, 24, 48, and 72 h taking aliquots of 1 mL from each tube. After appropriate dilutions with 0.9% NaCl, the samples were plated on Pseudomonas Agar Base (PAB, Oxoid), added with cetrimide fucidin cephaloridine (CFC) selective supplement and incubated at 25 °C for 48 h. All analyses were performed twice on two different samples.

### 2.4. Microbiological Analyses and pH Evaluation

The microbiological analyses were carried out not only on the burgers but also on the powder because peel and pulp dehydration at 37 °C took about a week, and therefore a possible contamination occurred. To the aim, 10 g of powder and 20 g of burger were aseptically transferred in sterile stomacher bags, diluted with 0.9% NaCl solution and homogenized with Stomacher LAB Blender 400 (Pbi International, Milan, Italy). Subsequently, decimal dilutions of homogenate samples were conducted using the same diluent and the dilutions were plated on appropriate media in Petri dishes. In particular, for the fish burgers, the media and the conditions used were: Plate Count Agar (PCA, Oxoid) incubated at 30 °C for 48 h and 5 °C for 10 days for the enumeration of mesophilic and psychrotrophic bacteria, respectively; Pseudomonas Agar Base (PAB, Oxoid), added with cetrimide fucidin cephaloridine (CFC) selective supplement and incubated at 25 °C for 48 h to enumerate *Pseudomonas* spp.; pour plated Iron Agar (IA) incubated at 25 °C for 3 days, for hydrogen sulfide producing bacteria (HSPB); pour plated IA, supplemented with 5 g/L NaCl and incubated at 15 °C for 7 days, for psychrotolerant and heat-labile aerobic bacteria (PHAB); Violet Red Bile Glucose Agar (VRBGA, Oxoid) incubated at 37 °C for 24 h for *Enterobacteriaceae*; de Man Rogosa Sharpe Agar (MRS, Oxoid), supplemented with cycloheximide (0.1 g/L Sigma) incubated at 37 °C for 48 h for Lactic Acid Bacteria (LAB). For the active powder the bacteria and the conditions used for their count were the same used for the burgers, except the PHAB detection because this microbial group was searched for exclusively in the fish products.

The pH of each homogenized sample was measured by a pH meter (Crison, Barbellona, Spain), after appropriate calibration.

### 2.5. Color Evaluation

Instrumental color readings of the prepared fish burgers were measured with Chromameter CR-400 colorimeter (Minolta Chromameter, Japan) after appropriate calibration, using a reference white tile. The color measurements were described in terms of Lightness (L*), redness (a*) and yellowness (b*) space values. Color measurements were made on the surface of each sample. For all investigated burgers four random readings were performed.

### 2.6. Sensory Evaluation

The sensory evaluation was performed by seven experienced panelists, researchers of the University of Foggia, selected several years prior to this research for their sensory skills to estimate fish attributes. For the current study, in a 3-h session, panelist reliability was assessed and sensory parameters to be taken into account were defined. Since sensory parameters could be differently perceived on raw and cooked burgers, the sensory evaluation was conducted on both, as also reported in other study [23]. Therefore, at each sampling time (0, 2, 5, 7, 9, 12, 14, 16), the CNT and the three active cod burgers (ACT-2.5; ACT-7.5 and ACT-12.5) were cooked in an electric convention oven (H2810, Hugin, Milan, Italy) at 180 °C for 20 min. For the analysis, each sample, both raw and cooked, was codified with three-digit code and offered to the panelists in individual cabin under controlled conditions of light, temperature and humidity. The panelists were asked to evaluate the color, odor, texture, and then to give an overall quality judgment of the fish burgers. The texture was judged by considering the force exerted for cutting the product with a knife [16]. A 9-point scale was used to quantify each attribute, where a score of 9 corresponded to “very good quality”, 7–8 to “good quality” and 6 to “sufficient quality”. The value of 5 represented the acceptability threshold, while scores from 4 to 1 corresponded to “unacceptable quality”.

### 2.7. Statistical Analysis

Tests were performed on duplicate batches. All experimental data are the average of three replicates. The results are presented as means ± Standard Deviation (SD) and graphically reported. Statistical significance was determined by one-way analysis of variance (ANOVA). Duncan’s multiple range test, with the option of homogeneous groups (*p* ≤ 0.05), was performed to determine significant differences among samples. To the aim, STATISTICA 7.1 for Windows (StatSoft, Inc., Tulsa, OK, USA) was used.

## 3. Results and Discussion

### 3.1. Effects of Prickly Pear Powder on Pseudomonas spp. by In Vitro Test

A preliminary in vitro test was performed on target bacteria (*Pseudomonas* spp.), before testing the active powder on fish burgers. The results are shown in Table 2. As it can be seen, there is a significant difference between the control sample and the active solutions, already after 24 h from the microbial inoculation. In particular, from the above mentioned data, it can be inferred that the Ctrl had a steady growth during all 72 h of the test, whereas for the active samples a reduction of the *Pseudomonas* spp. viable cell concentration was observed. It is worth noting that there is no difference between the investigated active samples, neither in terms of quantity used (i.e., 2.5% or 5%) nor in terms of the type of by-product (i.e., peel or pulp powder). As a matter of fact, all the concentrations chosen for the in vitro test showed a substantial antimicrobial activity on the target bacteria already after one day.

### 3.2. Microbial Contamination of Prickly Pear Powder

A preliminary microbiological analysis on the powder was carried out to assess whether prolonged dehydration at low temperature could have affected the microbiological stability. The results of this test are reported in Table 3. It can be noticed that for both the peel and pulp powders, only psychrotrophic bacteria were absent. The total mesophilic count (MES) and the lactic acid bacteria (LAB) recorded higher values compared to the other investigated microorganisms. Therefore, these results suggest that prickly pear dehydration at 37 °C promoted spoilage growth, most probably because the process took about one week. Due to the recorded powder contamination, antimicrobial effects of powder on fish burgers were assessed by comparing growth rate of main spoilage microorganisms, instead of using the values of specific viable cell concentrations.

### 3.3. Effects of Prickly Pear Powder on Microbiological Quality of Cod Fish Burgers

As reported in Section 2, the effects of prickly pear powder (i.e., 2.5 g, 7.5 g, and 12.5 g) on the microbial quality decay of fish burgers during refrigerated storage were assessed. Spoiled fish products are characterized by development of fishy, rotten H_2_S off-odors and unpleasant flavors, due to growth of specific spoilage microorganisms [25]. It is important to highlight that seafood deterioration is mainly caused by high microbial growth, that consequently provokes unpleasant chemical compounds production [26]. For this reason, *Pseudomonas* spp., hydrogen sulfide producing bacteria (HSPB), psychrotolerant and heat-labile aerobic bacteria (PHAB) were monitored as main spoilage groups.

**Figure 1:**
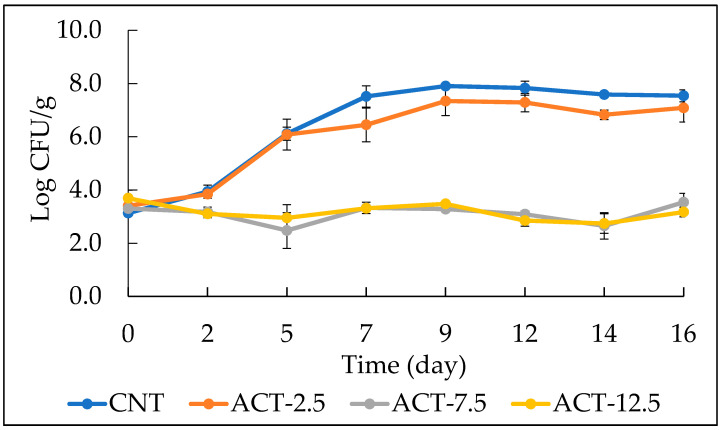
The evolution of *Pseudomonas* spp. viable cell concentration in fish burgers during 16 days of storage at 4 °C. Data indicate means ± SD. CNT: fish burger without prickly pear powder; ACT-2.5: fish burger enriched with 2.5 g of prickly pear powder; ACT-7.5: fish burger enriched with 7.5 g of prickly pear powder; ACT-12.5: fish burgers enriched with 12.5 g of prickly pear powder.

Figure 1 shows the evolution of *Pseudomonas* spp. viable cell concentration of the investigated fish burgers (i.e., CNT, ACT-2.5, ACT-7.5, and ACT-12.5). As it may be seen, during 16 days of storage, a decrease in the viable cell concentration (negative growth rate) was observed for the two samples ACT-7.5 and ACT-12.5. Despite the initial contamination, the addition of 7.5 g and 12.5 g of prickly pear powder had a significant effect on the growth of *Pseudomonas* spp., confirming data from the in vitro test (see Table 2). As one would expect, the CNT sample showed a rapid increase of microbial load during the first week of storage, which remained quite stable up to the end of the observation period. As shown in Figure 1, the ACT-2.5 sample had a trend similar to CNT, thus suggesting that at the lowest concentration, prickly pear powder had a minimal antimicrobial effect on *Pseudomonas* spp. proliferation. These results are in accordance with those of Ennouri et al. [27], who proved the antibacterial activity of the extract from prickly pear against *P. aeruginosa*. Furthermore, Palmeri et al. [12] also found that the water-based extract of prickly pear was effective in reducing the count of *Pseudomonas* spp. on sliced beef.

**Figure 2:**
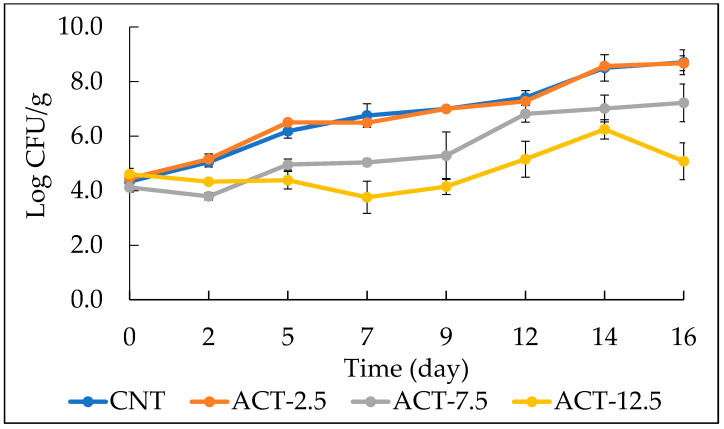
The evolution of total psycrhrotrophic bacteria in fish burgers during 16 days of storage at 4 °C. Data indicate means ± SD. CNT: fish burger without prickly pear powder; ACT-2.5: fish burger enriched with 2.5 g of prickly pear powder; ACT-7.5: fish burger enriched with 7.5 g of prickly pear powder; ACT-12.5: fish burgers enriched with 12.5 g of prickly pear powder.

Figure 2 exhibits the evolution of total psychrotrophic bacterial load during refrigerated storage. As can be seen, ACT-12.5 had shown the best antimicrobial effect throughout the storage period. It is worth noting that, despite powder contamination (see Table 3), a significant antimicrobial efficacy was found. In fact, a slight decrease in the viable cell concentration, followed by a gradual increase, was observed for the ACT-12.5 fish burger. The CNT, the ACT-2.5 and the ACT-7.5 samples have had a steady growth throughout the entire observation period. Among these last three samples, ACT-7.5 showed the best antimicrobial effect (i.e., the lowest growth rate), whereas ACT-2.5 and CNT showed a similar trend. These results suggest that prickly pear powder is not able to slow down the growth of psychrotrophic bacteria if used at low concentrations.

To make a comparison with literature, it must be observed that the results obtained in our experimental plan for the CNT sample are similar to those found in other studies dealing with fresh fish burgers [23,28], whereas, as regard active samples, Panza et al. [29] can be cited because these authors obtained a similar trend of psychrotrophic bacteria using pomegranate by-products to extend the shelf life of breaded cod sticks.

**Figure 3:**
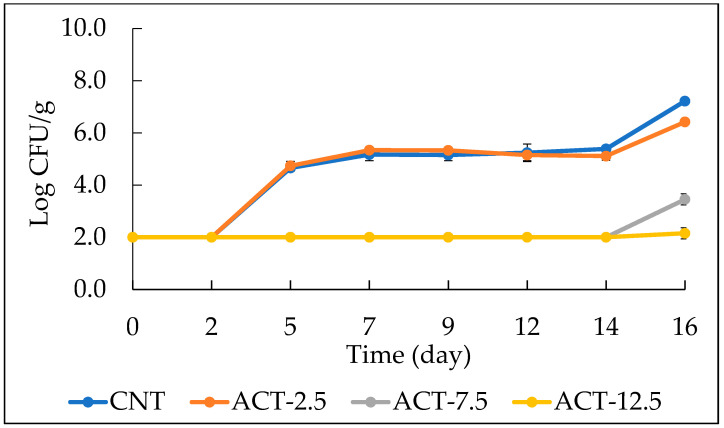
The evolution of psychrotolerant and heat-labile aerobic (PHAB) viable cell concentration in fish burgers during 16 days of storage at 4 °C. Data indicate means ± SD. CNT: fish burger without prickly pear powder; ACT-2.5: fish burger enriched with 2.5 g of prickly pear powder; ACT-7.5: fish burger enriched with 7.5 g of prickly pear powder; ACT-12.5: fish burgers enriched with 12.5 g of prickly pear powder.

The PHAB viable cell concentration plotted as a function of storage time is shown in Figure 3 for all fish burgers investigated in this study. The highest powder amount (12.5 g of active powder) was proven to be the most effective against this spoilage group. As a matter of fact, ACT-12.5 showed any microbial growth throughout the refrigerated storage. In the case of ACT-7.5 sample the microbial detection highlighted the same effects recorded in the previous sample (ACT-12.5), but there was a slight increase only on the last day of storage. The figure also shows that the CNT and the ACT-2.5 presented a very comparable trend, with a visible and rapid microbial growth from the second day of storage up to the end of the observation period.

**Figure 4:**
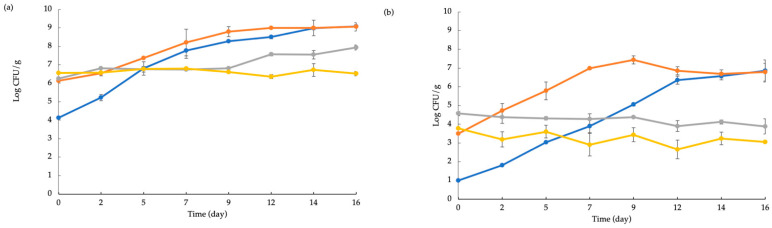

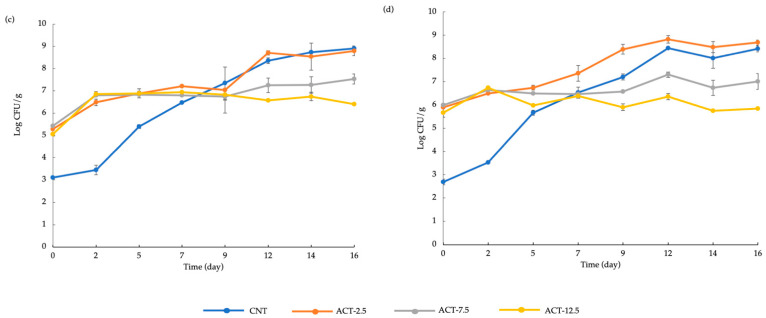
The evolution of total mesophilic bacteria (**a**) *Enterobacteriaceae* (**b**) Lactic acid bacteria (**c**) and Hydrogen Sulfide producing bacteria (**d**) in fish burgers during 16 days of storage at 4° C. Data indicate the mean ± SD. CNT: fish burger without prickly pear powder; ACT-2.5: fish burger enriched with 2.5 g of prickly pear powder; ACT-7.5: fish burger enriched with 7.5 g of prickly pear powder; ACT-12.5: fish burgers enriched with 12.5 g of prickly pear powder.

Figure 4 shows the microbial load evolution during the storage of the following groups: total mesophilic bacteria (Figure 4a), *Enterobacteriaceae* (Figure 4b), LAB (Figure 4c) and HSPB (Figure 4d). Despite the aforementioned powder contamination (see Table 3), for all the bacterial groups the antimicrobial effect of the prickly pear powder is evident when the growth rate of each sample is taken into account. As one would expect, the ACT-12.5 sample had the best antimicrobial effect considering that its growth rate was negligible and in some cases it was negative. Although the antimicrobial activity of the ACT-7.5 sample was evident, it was found to be slightly lower than that observed for the ACT-12.5 fish burger. For both CNT and ACT-2.5 a rapid increase in viable cell concentration was observed, visible for each microbial group (a–d). Regarding the growth rate, the ACT-2.5 sample had a lower value if compared to CNT, thus suggesting that also at the lowest concentration, prickly pear powder exerted a slight antimicrobial effect on these microbial groups. Similar trends were found by Nisar et al. [30], who used rosemary oil as natural preservative for bream fillets.

### 3.4. Effects of Prickly Pear Powder on pH of Cod Fish Burgers

**Figure 5:**
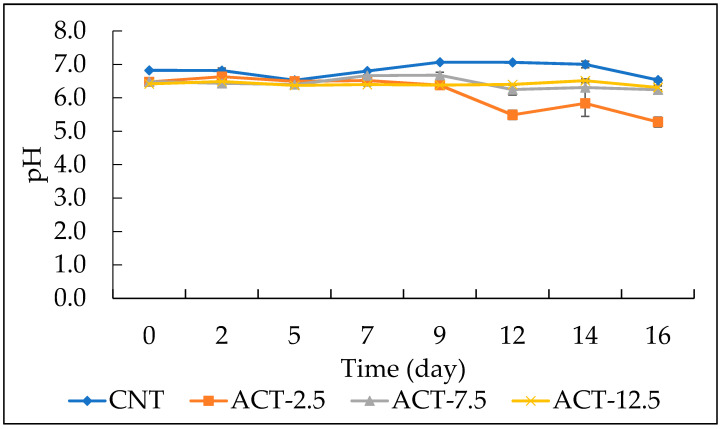
Trend of pH values of fish burgers during 16 days of storage at 4 °C. Data indicate means ± SD. CNT: fish burger without prickly pear powder; ACT-2.5: fish burger enriched with 2.5 g of prickly pear powder; ACT-7.5: fish burger enriched with 7.5 g of prickly pear powder; ACT-12.5: fish burgers enriched with 12.5 g of prickly pear powder.

The evolution during refrigerated storage of fish burger pH was reported in Figure 5. Initially, pH was similar for all the examined samples (around 7), in accordance with values also reported by other authors [28,31,32]. During time, the pH of CNT, ACT-7.5, and ACT-12.5 samples was almost constant throughout the storage period (6.53, 6.24 and 6.31 respectively), although the values of the two active burgers were slightly lower than those of the CNT. As long as the ACT-2.5 sample is concerned, pH values slightly decreased during time, most probably due to the high microbial proliferation occurred in this samples, in particular for the high lactic acid bacteria proliferation [33,34].

### 3.5. Effects of Prickly Pear Powder on Sensory Quality of Cod Fish Burgers

The scores for color, odor, and texture at the first and the last day of storage are presented in Table 4, for both raw and cooked fish burgers. Adding prickly pear powder, an aromatic and pleasant odor was perceived by the panelists, associated to the volatile compounds of prickly pears [7,8,9,10,11]. This perception was observed from the beginning and up to the last day of storage, for both raw and cooked fish products. These results are proven by the scores obtained from the panel, which were above the acceptability threshold (score = 5). Moreover, for ACT-7.5 and ACT-12.5, the presence of the powder led to the detection of any kind of fish odor, even at the last day of storage. An opposite behavior was found for the CNT samples that showed a high level of off-odors, especially on the 16th day of storage. Regarding the texture, in all the investigated samples, the values decreased during the storage period, thus proving that hardness and structure of the fish were lost during time. However, a statistically significant effect (*p* > 0.05) of prickly pear powder was observed. In particular, as the powder increased, the detrimental effect of storage time on the sample texture was less evident. This implies that the use of the powder could slow down texture decay during time. As long as the color attribute is concerned, a trend similar to that of the other two sensory attributes was observed. In particular, on the last day of storage, CNT sample recorded the worst score, ACT-2.5 was slightly better than the CNT, ACT-7.5 and ACT-12.5 obtained the best scores, and the differences between them were not statistically significant (*p* < 0.05).

The color change of the investigated fish burgers was also assessed by the colorimeter. The results are listed in Table 5, where the values are expressed as lightness (L*), redness (a*), and yellowness (b*). Data in Table 5 highlight that addition of prickly pear powder reduced the lightness of the burgers if compared to the CNT samples. Looking at the first column of Table 5, it can be also observed that lightness decreased as the powder concentration increased. This finding is not surprising, considering the dark color of the powder. In terms of the effects of time on color parameters, no great influence can be underlined. From data it’s possible to infer that lightness only in some cases slightly rose over storage, specifically for ACT-2.5 and ACT-7.5 [28].

The redness parameter (a*) was completely absent in CNT burgers during the entire storage period, while for the active samples, there was a significant reduction between the first and the last day of storage, more marked for the ACT-12.5. This color modification is caused by the migration of the prickly pear pigments from the powder to the minced cod fillets [35].

As far the yellowness parameter is concerned (b*), there were slight differences between CNT and active samples at both the beginning and at the end of the observation period. An increase in the b* value was observed for all samples between zero and 16 days of storage, thus demonstrating that fish decay is strictly linked to changes in yellowness parameters, regardless the by-product concentration. As a result, the use of the powder significantly affected the visual quality of fish burgers, as confirmed from data recorded on the color parameter during the panel test.

**Figure 6:**
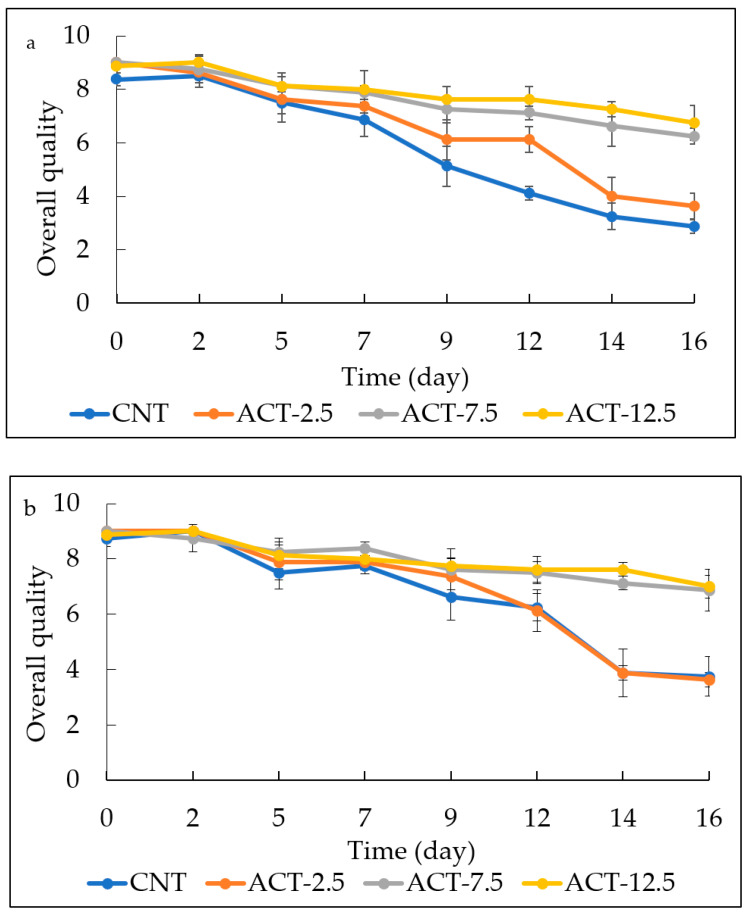
Evolution of Overall quality of both raw (**a**) and cooked (**b**) fish burgers during 16 days of storage. Data indicate means ± SD. CNT: fish burger without prickly pear powder; ACT-2.5: fish burger enriched with 2.5 g of prickly pear powder; ACT-7.5: fish burger enriched with 7.5 g of prickly pear powder; ACT-12.5: fish burgers enriched with 12.5 g of prickly pear powder.

The evolution of fish burger overall quality during 16 days of storage is shown in Figure 6a,b. A more pronounced reduction of the fish burger sensory quality was observed for the investigated raw samples, being the decay of the raw products faster than that of the cooked ones. As can be inferred from data shown in both graphs of Figure 6, CNT and ACT-2.5 had a similar trend, and became unacceptable during time. On the other hand, ACT-7.5 and ACT-12.5 samples showed good overall quality throughout the entire observation period and after two weeks these samples were found still completely acceptable. This may be due to the antimicrobial effect exerted by prickly pear powder, and in particular to the aromatic compounds that preserved the odor. Therefore, it is worth noting that the prickly pear powder added to the fish burgers did not worsen sensory quality, rather it improved the fish characteristics, especially in the raw samples (Figure 6a). In fact, already at the beginning of the sensory evaluation (time 0), the panelists appreciated the active samples more than the control. To sum up, the findings previously recorded in Table 4, dealing with specific sensory attributes, are in accordance with data on the general acceptance of the products shown in Figure 6.

## 4. Conclusions

The current study aimed to improve the storability of refrigerated cod fish burgers in accordance with the zero-waste concept. In fact, all the prickly pear parts were dehydrated, reduced in powder, and then used as food additives in the fish formulation, in a proportion that respected the zero-waste approach. An in vitro test was first run to assess the antimicrobial efficacy of this fruit powder. The results showed that both the concentrations chosen (2.5 and 5%) in this preliminary test had a marked antimicrobial activity on target bacteria, already after 24 h. The prickly pear powder was therefore added at three different amounts (2.5 g, 7.5 g and 12.5 g) to cod fish burgers, that were stored at 4 °C to assess powder efficacy in slowing down microbial growth. The results showed that the lowest prickly pear powder concentration used in this study did not affect the microbial growth at a great extent. Conversely, the other two tested concentrations, and especially the highest one, had a remarkable effect on the growth kinetic of spoilage microorganisms. In fact, in most cases a reduction of the growth rate was observed, if compared to the control sample. In few cases the growth rate was close to zero (i.e., total mesophilic bacteria and HSPB), and in one case (i.e., *Enterobacteriaceae*) a decrease in the viable cell concentration (negative growth rate) during refrigerated storage was observed. As long as the sensory quality decay is concerned, the results obtained in this study indicated that the prickly pear powder at two highest concentrations significantly slowed down the sensory quality decay during time of both raw and cooked cod fish burgers. This is most probably due to the antimicrobial activity of the powder and its aromatic compounds that improved the perceived odor. As a fact, the two burgers with 7.5 g and 12.5 g powder addition remained completely acceptable during the entire observation period. To sum up, due to the recorded results, the recycle of prickly pears seems interesting. The research needs to further explore the topic in terms of energy costs, emissions, etc., because drying of prickly pears is an energy-intensive operation. The use of renewable energy could be a starting point because companies are increasingly moving towards the use of green energy. Certainly, more in-depth studies would be needed for recommending zero-waste as a beneficial approach.

## Figures and Tables

**Table 1:** Formulation of the cod fish burgers with and without prickly pear powder addition.

Ingredients	CNT		ACT-2.5		ACT-7.5		ACT-12.5	
	Weight [g]	Weight [%]	Weight [g]	Weight [%]	Weight [g]	Weight [%]	Weight [g]	Weight [%]
Cod fillet	36.98	73.9	36.98	70.4	36.98	64.3	36.98	59.1
Extra-virgin olive oil	4.62	9.2	4.62	8.8	4.62	8.0	4.62	7.4
Potato starch	4.62	9.2	4.62	8.8	4.62	8.0	4.62	7.4
Potato flakes	3.7	7.4	3.7	7.0	3.7	6.4	3.7	5.9
Salt	0.1	0.2	0.1	0.2	0.1	0.2	0.1	0.2
Prickly pear powder	-	-	2.5	4.8	7.5	13.0	12.5	20.0

**Table 2:** Evolution of the *Pseudomonas* spp. viable cell concentration after inoculation of control and prickly pear peel (2.5% and 5%) and pulp (2.5% and 5%) powder. Data indicate means ± SD.

	log CFU/mL				
Sample Time (h)	0	4	24	48	72
Ctrl	2.96 ± 0.11	3.46 ± 0.09	8.82 ± 0.39	9.41 ± 0.17	9.49 ± 0.23
Peel 2.5%	3.12 ± 0.01	3.62 ± 0.09	1.30 ± 0.43	<10	<10
Peel 5%	3.26 ± 0.08	4.05 ± 0.14	2.09 ± 0.12	<10	<10
Pulp 2.5%	3.57 ± 0.21	3.80 ± 0.14	1.35 ± 0.49	<10	<10
Pulp 5%	3.51 ± 0.01	3.54 ± 0.09	2.76 ± 0.09	<10	<10

**Table 3:** Contamination of prickly pear peel and pulp powder (log CFU/g).

Sample	PSY	MES	PSE	ENTER	LAB	SHPB
Peel	-	7.14 ± 0.20	3.43 ± 0.10	4.71 ± 0.11	7.07 ± 0.10	6.02 ± 0.53
Pulp	-	7.03 ± 0.04	3.56 ± 0.04	3.35 ± 0.16	7.12 ± 0.16	5.15 ± 0.27

Data indicate means ± SD. PSY = Total Psycrhrotrophic bacteria < 10^2^ CFU/g; MES = Total Mesophilic bacteria; PSE = *Pseudomonas* spp.; ENTER = Enterobacteriaceae; LAB = Lactic Acid Bacteria; SHPB = Hydrogen Sulfide producing bacteria.

**Table 4:** Scores of sensory attributes (color, odor and texture) of raw and cooked fish burgers at 0 and 16 days of storage.

Samples		Storage Time (day)			
		Raw Fish Burger		Cooked Fish Burger	
		0	16	0	16
Color	CNT	8.1 ± 0.25 ^b^	2.9 ± 0.25 ^c^	8.3 ± 0.29 ^b^	4.1 ± 0.25 ^b^
	ACT-2.5	9.0 ± 0.10 ^a^	4.5 ± 0.58 ^b^	9.0 ± 0.10 ^a^	4.8 ± 0.29 ^b^
	ACT-7.5	9.0 ± 0.10 ^a^	6.1 ± 0.48 ^a^	9.0 ± 0.10 ^a^	6.3 ± 0.87 ^a^
	ACT-12.5	9.0 ± 0.10 ^a^	6.4 ± 0.75 ^a^	9.0 ± 0.10 ^a^	7.0 ± 0.41 ^a^
Odor	CNT	9.0 ± 0.10 ^a^	2.4 ± 0.25 ^d^	9.0 ± 0.10 ^a^	3.5 ± 0.58 ^b^
	ACT-2.5	9.0 ± 0.10 ^a^	3.6 ± 0.48 ^c^	9.0 ± 0.10 ^a^	3.6 ± 0.25 ^b^
	ACT-7.5	9.0 ± 0.10 ^a^	6.0 ± 0.29 ^b^	9.0 ± 0.10 ^a^	6.4 ± 0.48 ^a^
	ACT-12.5	9.0 ± 0.10 ^a^	6.9 ± 0.25 ^a^	9.0 ± 0.10 ^a^	7.0 ± 0.41 ^a^
Texture	CNT	8.1 ± 0.25 ^b^	3.6 ± 0.25 ^c^	8.3 ± 0.29 ^a^	4.0 ± 0.41 ^c^
	ACT-2.5	8.9 ± 0.25 ^a^	5.8 ± 0.50 ^b^	8.8 ± 0.29 ^a^	4.8 ± 0.29 ^b^
	ACT-7.5	9.0 ± 0.10 ^a^	6.5 ± 0.41 ^a,b^	8.9 ± 0.25 ^a^	7.0 ± 0.41 ^a^
	ACT-12.5	8.9 ± 0.25 ^a^	7.0 ± 0.71 ^a^	8.6 ± 0.48 ^a^	7.3 ± 0.29 ^a^

^a–d^ Data indicate means ± SD. For each sensory attribute, the values marked with different superscript letters in the column are significantly different (*p* < 0.05). CNT: fish burger without prickly pear powder; ACT-2.5: fish burger enriched with 2.5 g of prickly pear powder; ACT-7.5: fish burger enriched with 7.5 g of prickly pear powder; ACT-12.5: fish burgers enriched with 12.5 g of prickly pear powder.

**Table 5:** The effect of prickly pear enrichment on the color parameters of fish burgers.

Samples	L*		a*		b*	
	0	16	0	16	0	16
CNT	67.73 ± 1.87 ^a,A^	68.68 ± 1.97 ^a,A^	−2.79 ± 0.81 ^b,A^	−2.42 ± 0.78 ^d,A^	16.48 ± 1.43 ^b,B^	18.12 ± 1.64 ^c,A^
ACT-2.5	52.33 ± 1.56 ^b,B^	57.07 ± 1.64 ^b,A^	11.70 ± 1.78 ^a,A^	5.69 ± 0.59 ^c,B^	22.07 ± 1.80 ^a,B^	27.63 ± 1.62 ^a,A^
ACT-7.5	43.02 ± 1.04 ^c,B^	46.76 ± 1.70 ^c,A^	14.14 ± 1.95 ^a,A^	9.15 ± 0.68 ^a,B^	17.32 ± 1.41 ^b,B^	22.41 ± 1.11 ^b,A^
ACT-12.5	41.73 ± 0.39 ^c,A^	42.50 ± 1.66 ^d,A^	14.45 ± 0.73 ^a,A^	7.89 ± 0.58 ^b,B^	15.29 ± 0.81 ^b,A^	16.42 ± 1.32 ^c,A^

Data indicate means ± SD. Values marked with different superscript letters (a–d) in the column and superscript uppercase letters (A, B) in the row are significantly different (*p* < 0.05). CNT: fish burger without prickly pear powder; ACT-2.5: fish burger enriched with 2.5 g of prickly pear powder; ACT-7.5: fish burger enriched with 7.5 g of prickly pear powder; ACT-12.5: fish burgers enriched with 12.5 g of prickly pear powder. L* = lightness (0 = darkness, 100 = lightness); a* = redness (+60 = red, −60 = green); b* = yellowness (+60 = yellow, −60 = blue).

## Data Availability

The raw data will be made available upon request.

## References

[1] Aka S., Buyukdag N. (2021). How to prevent food waste behaviour? A deep empirical research. J. Retail. Consum. Serv..

[2] Song Q., Li J., Zeng X. (2015). Minimizing the increasing solid waste through zero waste strategy. J. Clean. Prod..

[3] Kumar K., Srivastav S., Sharanagat V.S. (2021). Ultrasound assisted extraction (UAE) of bioactive compounds from fruit and vegetable processing by-products: A review. Ultrason. Sonochem..

[4] Dilucia F., Lacivita V., Conte A., Del Nobile M.A. (2020). Sustainable Use of Fruit and Vegetable By-Products to Enhance Food Packaging Performance. Foods.

[5] Karimi A., Kazemi M., Amiri Samani S., Simal-Gandara J. (2021). Bioactive compounds from by-products of eggplant: Functional properties, potential applications and advances in valorization methods. Trends Food Sci. Technol..

[6] Andreu-Coll L., Cano-Lamadrid M., Sendra E., Carbonell-Barrachina Á., Legua P., Hernández F. (2019). Fatty acid profile of fruits (pulp and peel) and cladodes (young and old) of prickly pear [*Opuntia ficus-indica* (L.) Mill.] from six Spanish cultivars. J. Food Compos. Anal..

[7] Bouazizi S., Montevecchi G., Antonelli A., Hamdi M. (2020). Effects of prickly pear (*Opuntia ficus-indica* L.) peel flour as an innovative ingredient in biscuits formulation. LWT Food Sci. Technol..

[8] Cardador-Martínez A., Jimenez Martinez C., Sandoval G. (2011). Revalorization of cactus pear (*Opuntia* spp.) wastes as a source of antioxidants. Food Sci. Technol..

[9] Palmeri R., Parafati L., Arena E., Grassenio E., Restuccia C., Fallico B. (2020). Antioxidant and antimicrobial properties of semi-processed frozen prickly pear juice as affected by cultivar and harvest time. Foods.

[10] Karabagias V.K., Karabagias I.K., Prodromiti M., Gatzias I., Badeka A. (2020). Bio-functional alcoholic beverage preparation using prickly pear juice and its pulp in combination with sugar and blossom honey. Food Biosci..

[11] Valero-Galván J., González-Fernández R., Sigala-Hernández A., Núnez-Gastélum J.A., Ruiz-May E., Rodrigo-García J., Larqué-Saavedra A., del Rocío Martínez-Ruiz N. (2021). Sensory attributes, physicochemical and antioxidant characteristics, and protein profile of wild prickly pear fruits (*O. macrocentra* Engelm., *O. phaeacantha* Engelm., and *O. engelmannii* Salm-Dyck ex Engelmann.) and commercial prickly pear fruits (*O. ficus-indica* (L.) Mill.). Food Res. Int..

[12] Palmeri R., Parafati L., Restuccia C., Fallico B. (2018). Application of prickly pear fruit extract to improve domestic shelf life, quality and microbial safety of sliced beef. Food Chem. Toxicol..

[13] Melgar B., Dias M.I., Ciric A., Sokovic M., Garcia-Castello E.M., Rodriguez-Lopez A.D., Barros L., Ferreira I. (2017). By-product recovery of *Opuntia* spp. peels: Betalainic and phenolic profiles and bioactive properties. Ind. Crop. Prod..

[14] Chougui N., Djerroud N., Naraoui F., Hadjal S., Aliane K., Zeroual B., Larbat R. (2015). Physicochemical properties and storage stability of margarine containing Opuntia ficus-indica peel extract as antioxidant. Food Chem..

[15] Parafati L., Restuccia C., Palmeri R., Fallico B., Arena E. (2020). Characterization of prickly pear peel flour as a bioactive and functional ingredient in bread preparation. Foods.

[16] Danza A., Conte A., Del Nobile M.A. (2017). Technological options to control quality of fish burgers. J. Food Sci. Technol..

[17] Spinelli S., Conte A., Lecce L., Incoronato A.L., Del Nobile M.A. (2014). Microencapsulated propolis to enhance the antioxidant properties of fresh fish burgers. J. Food Proc. Eng..

[18] Parlapani F.F. (2021). Microbial diversity of seafood. Curr. Opin. Food Sci..

[19] Houicher A., Bensid A., Regenstein J.M., Ozogul F. (2021). Control of biogenic amine production and bacterial growth in fish and seafood products using phytochemicals as biopreservatives: A review. Food Biosci..

[20] Kontominas M.G., Badeka A.V., Kosma I.S., Nathanailides C.I. (2021). Innovative seafood preservation technologies: Recent developments. Animals.

[21] Olatunde O.O., Benjakul S. (2018). Natural preservatives for extending the shelf-life of seafood: A revisit. Compr. Rev. Food Sci. Food Saf..

[22] Hasani S., Ojagh S.M., Ghorbani M., Hasani M. (2020). Nano-encapsulation of lemon essential oil approach to reducing the oxidation process in fish burger during refrigerated storage. J. Food Biosci. Technol..

[23] Cedola A., Cardinali A., Del Nobile M.A., Conte A. (2017). Fish burger enriched by olive oil industrial by-product. Food Sci. Nutr..

[24] Del Nobile M.A., Corbo M.R., Speranza B., Sinigaglia M., Conte A., Caroprese M. (2009). Combined effect of MAP and active compounds on fresh blue fish burger. Int. J. Food Microbiol..

[25] Albertos I., Marrtin-Diana A.B., Burón M., Rico D. (2019). Development of functional bio-based seaweed (*Himanthalia elongata* and *Palmaria palmata*) edible films for extending the shelf life of fresh fish burgers. Food Packag. Shelf Life.

[26] Gram L., Dalgaard P. (2002). Fish spoilage bacteria—Problems and solutions. Curr. Opin. Biotechnol..

[27] Ennouri M., Ammar I., Khemakhem B., Attia H. (2014). Chemical composition and antibacterial activity of *Opuntia Ficus-Indica F. Inermis* (Cactus Pear) Flowers. J. Med. Food.

[28] Rico D., Albertos I., Martinez-Alvarez O., Lopez-Caballero M.E., Martin-Diana A.B. (2020). Use of sea fennel as a natural ingredient of edible films for extending the shelf life of fresh fish burgers. Molecules.

[29] Panza O., Conte A., Del Nobile M.A. (2021). Pomegranate by-products as natural preservative to prolong the shelf life of breaded cod stick. Molecules.

[30] Nisar T., Yang X., Alim A., Iqbal M., Wang Z., Guo Y. (2019). Physicochemical responses and microbiological changes of bream (*Megalobrama ambycephala*) to pectin-based coatings enriched with clove essential oil during refrigeration. Int. J. Biol. Macromol..

[31] Mexis S.F., Chouliara E., Kontominas M.G. (2009). Combined effect of an oxygen absorber and oregano essential oil on shelf life extension of rainbow trout fillets stored at 4 °C. Food Microbiol..

[32] Gao M., Feng L., Jiang T., Zhu J., Fu L., Yuan D., Li J. (2014). The use of rosemary extract in combination with nisin to extend the shelf life of pompano (*Trachinotus ovatus*) fillet during chilled storage. Food Control..

[33] Kitundu E., Young O., Seale B., Owens A. (2021). Lactic fermentation of cooked, comminuted mussel, *Perna canaliculus*. Food Microbiol..

[34] Danza A., Lucera A., Lavermicocca P., Lonigro S.L., Bavaro A.R., Mentana A., Centonze D., Conte A., Del Nobile M.A. (2018). Tuna Burgers Preserved by the Selected *Lactobacillus paracasei* IMPC 4.1 Strain. Food Bioprocess. Technol..

[35] Kharrat N., Salem H., Mrabet A., Aloui F., Triki S., Fendri A., Gargouri Y. (2018). Synergistic effect of polysaccharides, betalain pigment and phenolic compounds of red prickly pear (*Opuntia stricta*) in the stabilization of salami. Int. J. Biol. Macromol..

